# Evaluating the role of generative AI and color patterns in the dissemination of war imagery and disinformation on social media

**DOI:** 10.3389/frai.2024.1457247

**Published:** 2025-01-06

**Authors:** Estibaliz García-Huete, Sara Ignacio-Cerrato, David Pacios, José Luis Vázquez-Poletti, María José Pérez-Serrano, Andrea Donofrio, Clemente Cesarano, Nikolaos Schetakis, Alessio Di Iorio

**Affiliations:** ^1^Department of Journalism and Global Communication, Faculty of Information Sciences, Universidad Complutense de Madrid, Madrid, Spain; ^2^Optics Department, Faculty of Optics and Optometry, Universidad Complutense de Madrid, Madrid, Spain; ^3^Department of Computer Architecture and Automation, Faculty of Informatics, Universidad Complutense de Madrid, Madrid, Spain; ^4^Section of Mathematics, International Telematic University Uninettuno, Rome, Italy; ^5^Computational Mechanics and Optimization Laboratory, School of Production Engineering and Management, Technical University of Crete, Chania, Greece; ^6^Quantum Innovation Pc, Chania, Greece; ^7^Alma Sistemi Srl, Rome, Italy

**Keywords:** social media, disinformation, generative AI, color patterns, LUTs, fake news, war imagery, information manipulation

## Abstract

This study explores the evolving role of social media in the spread of misinformation during the Ukraine-Russia conflict, with a focus on how artificial intelligence (AI) contributes to the creation of deceptive war imagery. Specifically, the research examines the relationship between color patterns (LUTs) in war-related visuals and their perceived authenticity, highlighting the economic, political, and social ramifications of such manipulative practices. AI technologies have significantly advanced the production of highly convincing, yet artificial, war imagery, blurring the line between fact and fiction. An experimental project is proposed to train a generative AI model capable of creating war imagery that mimics real-life footage. By analyzing the success of this experiment, the study aims to establish a link between specific color patterns and the likelihood of images being perceived as authentic. This could shed light on the mechanics of visual misinformation and manipulation. Additionally, the research investigates the potential of a serverless AI framework to advance both the generation and detection of fake news, marking a pivotal step in the fight against digital misinformation. Ultimately, the study seeks to contribute to ongoing debates on the ethical implications of AI in information manipulation and to propose strategies to combat these challenges in the digital era.

## 1 Introduction

To contextualize this work, it is necessary to understand other previous conflicts. To do so, this introduction will analyze disinformation campaigns in other war conflicts, starting with how the Nazis used propaganda (Lasswell, 1951) as a weapon of war until the escalation of the conflict with World War II.

The Nazi propaganda during World War II depicted Jews as disease vectors, contaminants, and disgusting animals, and even used representations of meat to demonize them (Wit, 2021; Buscemi, 2016). Radio propaganda (Lasswell, 1951) further fueled anti-Semitic sentiments and encouraged expressions of hatred (Adena et al., 2015). The dehumanization of Jews in Nazi propaganda was a key element in the plan for their destruction (Brown, 2019). The invasion of Poland by the Wehrmacht marked the beginning of World War II, during which these propaganda (Lasswell, 1951) strategies were utilized to propagate anti-Semitic ideologies (Russell, 2018). During World War II, the Office of Strategic Services (OSS) utilized psychological operations, including the dissemination of misinformation, to achieve strategic aims without escalating into major conflicts (Wit, 2021). The global newsreel industry during World War II was heavily focused on war-related stories to the extent that domestic news unrelated to the war effort was overshadowed. This saturation of war-related content in newsreels demonstrates how information dissemination during World War II was influenced by the prevailing narratives of the time (Althaus, 2010).

These unethical practices explained in previous conflicts have been escalating to the present day where social networks are used as a means to spread fake news. As Guillamet (2018) posits, this manipulation has manifested in various forms, ranging from decontextualized to outright fabricated videos, photos, and statements. The conflict in Ukraine has highlighted the extent to which artificial intelligence, refining the techniques pioneered by Adobe's Photoshop, has been co-opted into the arsenal of propaganda. The integration of color pattern knowledge into generative AI technologies has enabled the production of war imagery so sophisticated that it might be indistinguishable from authentic photographs.

This paradigm shift requires increased vigilance in informational consumption, while paradoxically making manipulation and confrontation, economic, political, and ethical, more accessible, particularly on social networks such as the platform formerly known as Twitter. With Elon Musk's acquisition and subsequent updates to this platform, its role as a medium of communication in the strictest sense has been underscored. This article will examine the manipulated imagery distributed during the Ukraine-Russia conflict, evaluating both the use of Adobe tools and AI technologies and their subsequent social impact. The economic consequences are evident, as advertising has been fundamental to the economic viability of media outlets, leading to a symbiosis described (Vergara and Santibáñez, 2008) as a link between media dissemination and financial survival. Nevertheless, this has led to the prioritization of advertising objectives over journalistic ethics, thereby compromising the quality and integrity of journalism, the transformation of Twitter, especially under Musk's stewardship, into a platform for both media and advertising convergence is notable. The introduction and relaunch of Twitter Blue in 2022, which allows account verification and potential revenue generation, exemplifies this convergence. According to the Ads Revenue Sharing program, certain criteria, such as subscription tenure and tweet impressions, dictate the monetization potential, which has been exploited in the dissemination of misinformation, particularly in the context of the Russian-Ukrainian conflict. Furthermore, the exploitation of AI in image reproduction has not only facilitated the spread of misinformation but also incurred economic losses, as evidenced by the dissemination of a fabricated explosion near the Pentagon by Russian state media networks on X (formerly known as Twitter), initially propagated through Facebook.

From a normative perspective, the pursuit of objective journalism is intertwined with the authenticity of news imagery, an assertion central to the cultural authority of journalists as discussed by Carlson (2009). Technological advances in digital image processing have challenged existing norms, necessitating the establishment of clear standards within photojournalism. The application of color search tables (LUTs) can modify images to appear more realistic without altering the original content, emphasizing the importance of color tones in conveying messages in photography. Following the same path, manipulation of images for political gain and the dissemination of propaganda on social networks have contributed to a global climate of distrust in traditional pillars of social well-being, including political systems and media outlets, as well as in technology companies that exert control over digital services, as observed (Canals, 2022). The political use of image manipulation to discredit opponents, such as the deep-fake videos of President Zelensky of Ukraine, is symptomatic of broader strategies that exploit homophobia to dehumanize and “other” individuals, thus undermining their humanity and social standing, as elaborated by Cornejo Espejo (2012).

This study aims to answer the following questions:

- RQ1: How do color patterns (LUTs) influence the perceived authenticity of AI-generated war imagery in the context of digital disinformation?
- RQ2: What are the ethical, political, and social consequences of widespread use of generative AI technology for creating realistic fake images in war-related scenarios?
- RQ3: How effective is a serverless AI framework in generating and identifying fake news images, and what are its potential applications in countering disinformation campaigns?
- RQ4: To what extent can specific color adjustments and histograms be used to distinguish between authentic and AI-generated war photographs?

A comprehensive analysis is set to be constructed within this work, encompassing the evolution and influence of social media in the dissemination of disinformation from the Ukrainian conflict's inception to the present day. The progression from an era devoid of generative AI technologies to their current advanced implementations will be traced, with a focus on examining the relationship between color patterns (LUTs) in war imagery and their perceived authenticity. This scrutiny is expected to illuminate the complex interplay between the veracity of images and the perception of the viewer, as well as the extensive economic, political, and social consequences that surface when manipulative practices are brought to light.

Therefore, an experimental initiative is proposed to create and train a generative artificial intelligence (AI) model capable of producing war imagery that closely mimics real-life footage. The success of this experiment could establish a correlation between specific color patterns and the likelihood of images being perceived as authentic, providing new perspectives on the dynamics of misinformation. Moreover, the investigation of a serverless AI framework in this particular situation is predicted to represent noteworthy progress in the domain of fake news creation and identification. This strategy is anticipated to not only enhance the overall comprehension of disinformation campaigns but also serve as a precursor for forthcoming techniques aimed at countering the dissemination of fake news. Through conducting a comprehensive analysis and utilizing creative methodologies, the objective of this study is to make a substantial contribution to the ongoing discussion regarding the ethical consequences and possible countermeasures against the manipulation of information in the digital era.

Our contributions and findings are summarized in the following points:

- We create a system to produce artificial war photographs using AI. This system utilizes a serverless computing framework, specifically AWS Lambda, to effectively generate a substantial collection of fake images that mimic real war photos.
- Our analysis investigates the relationship between color patterns (LUTs) in war imagery and their perceived authenticity. By examining color histograms and variations, we provide insights into how specific color adjustments can enhance the realism of AI-generated images for viewers.
- We discuss the broader ethical, political, and social implications of AI-generated disinformation. Our study highlights the potential risks associated with the widespread availability of technology capable of creating realistic fake images, and underscores the need for effective detection methods and ethical guidelines.
- Our innovative serverless AI framework greatly enhances the production and detection of fake news images. This adaptable and economical framework supports the creation and examination of extensive image datasets, aiding in the comprehension and formulation of strategies to combat digital disinformation efforts.

The paper contains the following sections: Section 2 reviews the literature analyzed for the development of this research, Section 3 analyzes the factors that affect the generation of the photographs and reviews the LUTs, as well as the purpose of using histograms, Section 3.4 explain the methodology for the generalization of the images, Section 4 develops serverless technology and coordinates each of its elements for the generalization of images, Section 6 shows the conclussions related to this study and Section 5 discusses all the answer related to this study.

## 2 Related work

A review of the state of the art has been carried out on different works dealing with fake news, the use of deep fake, its influence in networks, as well as bibliography related to the field of colorimetry and serverless computing.

### 2.1 Fake news and misinformation content

A small bibliographic review of articles dealing with the subject of fake news and how it affects disinformation content in social networks was carried out. First, we will define the concept of information disorder Wardle and Derakhshan (2017) as a spectrum of false, misleading, or harmful information that is deliberately or inadvertently spread, including misinformation, disinformation, and malinformation, which distorts public perception and undermines trust in reliable sources. After introducing it, we will proceed to contextualize the context of fakes news and disinformative content.

Di Domenico et al. (2021) reviews the impact of fake news on marketing and consumer behavior, identifying five key themes: dissemination process, channel characteristics, results, fabricated legitimacy, and attitudes. It highlights the rapid spread of misinformation on social networks due to low entry barriers and sensationalist headlines, significantly affecting consumer perceptions and behaviors toward brands, leading to potential reputational and financial damage. The authors propose a theoretical framework for future research, stressing the need to address definitional issues of fake news, examine psychological and technological spread factors, and understand severe consequences for consumers and firms. They suggest developing strategies to combat fake news, improve consumer information literacy, and explore the role of social media platforms in perpetuating misinformation. Mubarak et al. (2023) investigates tweet deletions, focusing on Arabic tweets, revealing that harmful content such as hate speech and disinformation often leads to deletions, and highlights Twitter's role in suspending accounts. This research emphasizes the use of deleted tweets to develop models to detect and prevent harmful content. A¨ımeur et al. (2023) reviews fake news, disinformation, and misinformation on social media, focusing on the role of AI in both the creation and combating of fake news. It discusses various AI-based detection approaches, their limitations, and the need for improved algorithms, better fact-checker collaboration, and improved public awareness, calling for ongoing research to address fake news spread on social media.

As can be seen after conducting this small review of the literature, fake news is almost always linked to the use of AI, either to generate fake images or fake news. This content is posted on social networks, where it is impossible to verify its veracity, and from there are two options; either the content is removed or it spreads as news.

### 2.2 Generative AI in the context of the Russia–Ukraine war

After developing how this fake news influences, we will assess how the creation of this content affects the war context of the Russia–Ukraine war. For this purpose, a literature review will be carried out and here we will highlight the articles that have helped us in the development of generative AI.

Urman and Makhortykh (2023) investigates the handling of politically sensitive content and the tendency to disseminate fake news by language models (LLMs) such as ChatGPT, Google Bard, and Bing Chat, comparing their effectiveness in Russian, Ukrainian, and English. Bing Chat was the least likely to provide fake news, while Google Bard performed better in English than in Ukrainian or Russian, and ChatGPT showed superior performance in Ukrainian. Makhortykh et al. (2023), though all models struggled with factual accuracy regarding political figures' personal lives. The study emphasizes the need for robust mechanisms to detect and mitigate misinformation in LLMs, highlighting the importance of transparency and improved training to maintain public trust. Another study (Kuznetsova et al., 2023) evaluates the effectiveness of ChatGPT and Bing Chat in verifying political information on five contentious topics, using the AI audit methodology. ChatGPT showed higher accuracy (72%) compared to Bing Chat (67%), with significant performance variability based on language and topic, indicating biases and challenges in low-resource languages. Both studies underscore the need for comparative research to address biases, improve LLMs' effectiveness, and ensure transparent and equitable performance in diverse languages and topics.

As can be seen in this bibliography, this AI, due either to the training set or to the algorithm itself, gives somewhat ambiguous answers or generates somewhat ambiguous images of a conflict. It is very important to detect this type of elements in order to know what kind of news has been generated with AI.

### 2.3 Colorimetric analysis using histograms

Another branch of this article is colorimetric analysis; for this, it is important to understand how other types of analysis are performed with the histogram and why it is important in colorimetry.

Bueno et al. (2016) introduces a mobile, affordable fluorescence detection system designed to measure ochratoxin A (OTA) levels in drinks such as beer and wine. Utilizing a CMOS sensor paired with a MATLAB-based interface, this system captures and analyzes images of OTA-illuminated samples, taking advantage of OTA's inherent fluorescence when exposed to UV light. The study finds that RGB and HSV color models effectively correlate with OTA concentrations, allowing detection without extraction columns. Despite wine's color complexity, the device effectively differentiates OTA levels, though with less sensitivity than for beer. Compared to traditional methods like HPLC, the device offers advantages in cost, portability, and ease of use, suggesting potential for rapid, in situ food safety testing with further refinement.

Jalal et al. (2017) introduces a smartphone-based app for rapid, cost-effective colorimetric quantification of blood hematocrit levels. By integrating a microfluidic chip with a smartphone camera and a custom optical platform, the app converts color images of blood samples into grayscale histograms to determine hematocrit concentrations. Validated against standard techniques, the app shows comparable accuracy and reliability, providing a portable and easy-to-use alternative for point-of-care testing. Its affordability, minimal sample preparation, and rapid analysis make it suitable for resource-limited settings, with potential extensions to other biomarkers. Future enhancements, such as integrating machine learning, could further improve accuracy and usability, making it a powerful tool for real-time medical diagnostics.

These studies show how colorimetric analysis is an important asset when analyzing images. This type of analysis can be of interest to determine the veracity of AI-generated images.

### 2.4 Serverless computing

Then, literature review of serverless computing was also conducted. We will now comment on the most relevant articles.

The first article Pacios et al. (2023) investigates using a serverless computing architecture to process ionograms from the Mars Express mission's MARSIS instrument, addressing the unfeasible manual analysis of over 2 million noisy and complex entries. Using AWS Lambda, the suggested modular architecture drastically cuts down processing time and expenses, managing the complete dataset in just 20 minutes, a feat unachievable by local solutions. The system effectively identifies oblique echoes, which are essential for studying Mars' ionosphere, though it has a 25% false-positive rate primarily caused by cyclotron echoes. This initial dataset can train machine-learning models for further refinement, demonstrating serverless computing's potential for rapid, scalable, and cost-effective planetary data processing. The second article Pacios et al. (2024) applies a serverless architecture to detect Martian auroras using data from the Emirates Mars Mission, achieving high accuracy and efficiency with minimal resource consumption. This system, utilizing AWS Lambda and Amazon SageMaker, shows the advantages of serverless computing for real-time image analysis in planetary science. The third study Iacono et al. (2023) introduces SNDVI, a serverless framework to calculate the Normalized Difference Vegetation Index (NDVI) from large multispectral datasets using AWS Lambda. SNDVI processes images significantly faster than traditional methods, with a total cost of $4.19, demonstrating its efficiency and cost-effectiveness. The research highlights serverless computing's benefits for large-scale NDVI computations, suggesting future enhancements for broader agricultural applications.

As can be seen in the bibliography, both generative AI and serverless technology are groundbreaking technologies that have opened new horizons in various fields, including image processing and other areas. It is important to consider them for the development of this work.

## 3 LUTs and colometry theory

### 3.1 Color measuring applied to photography

**Figure 1:**
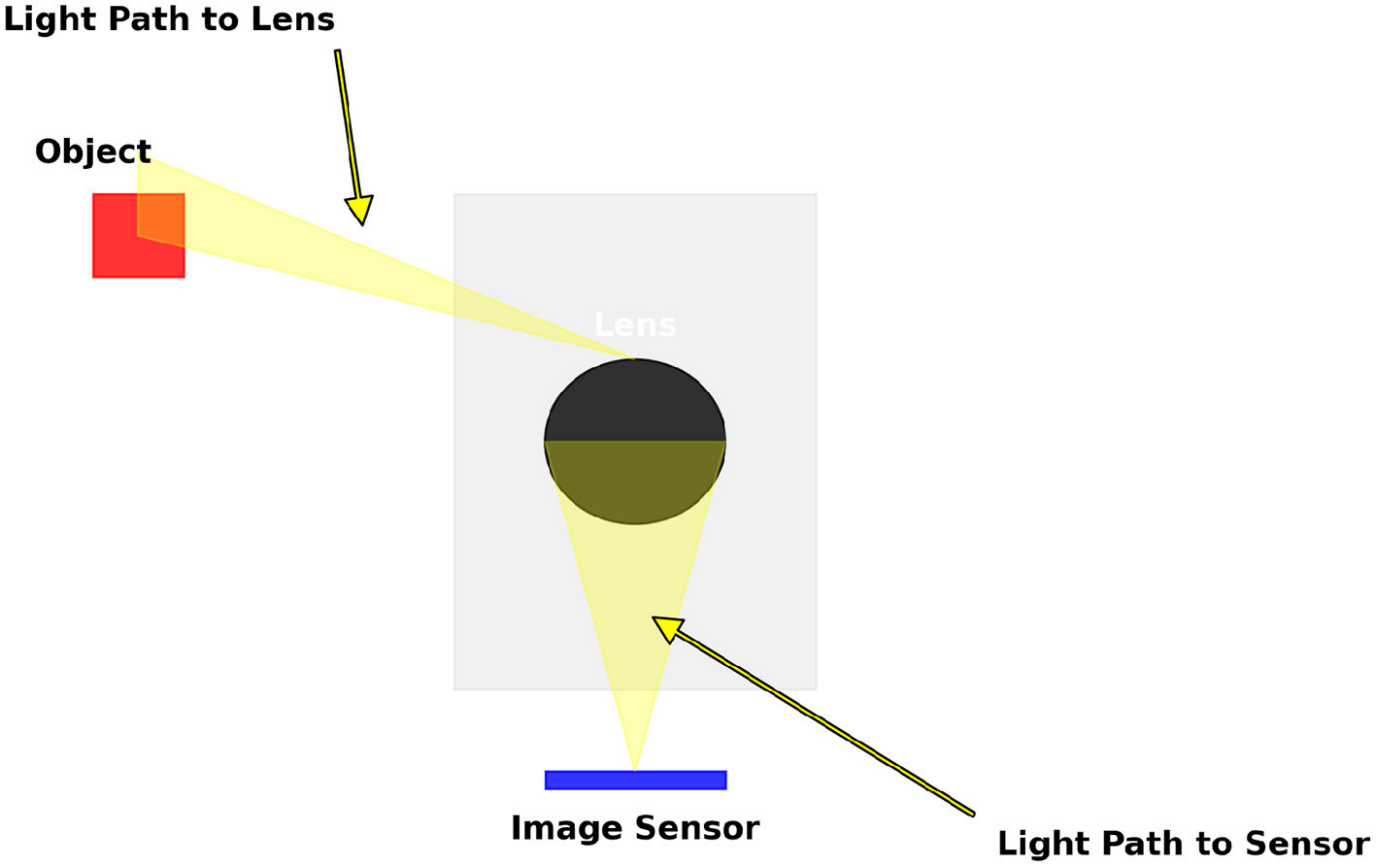
Scheme resuming the path of a photography: The light from the object enters the optics of the camera (lens) and finally arrives at the sensor where it's renderized.

When a photograph is taken, an image of an object is taken, it will first pass through the camera's optics (Mart́ınez-Verdú et al., 2010) and finally reach the camera's sensor. Where it will be processed and rendered for its final image. During this process (Figure 1), the color characteristics will change, since both the camera optics and the sensor resolution will influence the chromaticity of the colors. The chromacity can change through the following mechanism:

1. Lens characteristics (Bhatia et al., 2009): different lenses have distinct optical properties that significantly impact how colors are transmitted and focused onto the camera sensor. The materials used in lens construction, such as glass or specialized plastics, influence light transmission and color accuracy. High-quality glass typically provides better color fidelity and less distortion. Additionally, the design of a lens, including the number and arrangement of lens elements, plays a crucial role in determining its optical performance. Complex lens designs with multiple elements can correct various optical aberrations and improve color rendering. The focal length and aperture of a lens also affect color reproduction. Wide-angle lenses may introduce more color fringing and distortion at the edges, while telephoto lenses can enhance color contrast and saturation. Overall, the choice of lens and its specific characteristics directly influence the final color quality of the captured images, making it essential for photographers to select lenses that match their desired aesthetic and technical requirements.
2. Lens coatings (Subedi et al., 2018): modern lenses often feature advanced coatings designed to enhance image quality by reducing reflections, glare, and chromatic aberrations. These coatings, such as multi-layer anti-reflective coatings, are applied to the surface of lens elements to improve light transmission and minimize unwanted reflections. By reducing reflections, these coatings help maintain higher contrast and prevent flare, which can wash out colors and reduce image clarity. Additionally, lens coatings can affect the transmission and dispersion of different wavelengths of light, thereby influencing color saturation and fidelity. For instance, coatings designed to block ultraviolet (UV) light can prevent color casts caused by UV radiation. Moreover, some coatings are specifically engineered to enhance certain colors, providing more vibrant and accurate color reproduction. The effectiveness of lens coatings is particularly noticeable in challenging lighting conditions, such as shooting against the sun or in high-contrast scenes, where maintaining color accuracy is crucial for achieving high-quality photographs.
3. Chromatic aberration (Campbell and Gubisch, 1967): chromatic aberration is an optical phenomenon that occurs when different colors of light are focused at slightly different points by a lens, resulting in color fringing around high-contrast edges in an image. This effect is more pronounced in cheaper lenses or lenses with simpler optical designs. Chromatic aberration can be categorized into two types: longitudinal (axial) and lateral (transverse). Longitudinal chromatic aberration causes different wavelengths to focus at different distances from the lens, leading to color fringes along the depth of the image. Lateral chromatic aberration occurs when different wavelengths are focused at different positions on the image sensor, causing color fringing around the edges of the image. High-quality lenses use specialized glass materials and advanced optical designs to minimize these aberrations. Aspheric lens elements and low-dispersion glass are common solutions to reduce chromatic aberration. Effective correction of chromatic aberration is essential for achieving high color accuracy and sharpness in images, particularly in situations with strong color contrasts, such as landscape photography or macrophotography.
4. Filters (Khokhar et al., 2011): photographers often use filters over their lenses to achieve specific artistic effects or to correct for certain lighting conditions. Filters can significantly alter the color balance of an image by selectively blocking or transmitting certain wavelengths of light. There are various types of filters, each serving a different purpose. For example, polarizing filters reduce reflections and glare from non-metallic surfaces, enhancing color saturation and contrast in outdoor photography. Neutral density (ND) filters reduce the amount of light entering the lens without affecting color balance, allowing for longer exposures or wider apertures in bright conditions. Graduated ND filters are useful for balancing the exposure between the sky and the foreground in landscape photography. Color filters, such as warming or cooling filters, adjust the overall color temperature of the image, compensating for the color cast of different light sources. UV filters block ultraviolet light, preventing color casts and haze in outdoor photographs. The choice of filter can greatly impact the mood and tone of an image, providing photographers with creative control over the final appearance of their photos.
5. Aperture settings (Hasinoff and Kutulakos, 2011): the aperture of a lens controls the amount of light entering the camera and affects depth of field, which in turn influences the intensity and distribution of colors in an image. Aperture is measured in f-stops, with lower f-stop numbers indicating larger apertures that allow more light to reach the sensor. Larger apertures (e.g., f/1.8 or f/2.8) create a shallow depth of field, isolating the subject from the background and enhancing color contrast and saturation in the focused area. This effect is often used in portrait photography to create a pleasing bokeh and emphasize the subject. Conversely, smaller apertures (e.g., f/16 or f/22) increase depth of field, bringing more of the scene into sharp focus. However, very small apertures can cause diffraction, reducing overall sharpness and potentially affecting color clarity. Additionally, aperture settings can influence the lens's optical performance, with some lenses performing best at mid-range apertures (e.g., f/8 to f/11) in terms of sharpness and color accuracy. Understanding the interplay between aperture and color reproduction helps photographers make informed decisions to achieve their desired visual outcomes.
6. Sensor characteristics (Hain et al., 2007): while not directly related to optics, the sensor in a digital camera plays a crucial role in color reproduction. The type of sensor, such as Charge-Coupled Device (CCD) or Complementary Metal-Oxide-Semiconductor (CMOS), affects how colors are captured and processed. CCD sensors are known for their high image quality and color accuracy, but are typically more power-hungry and expensive. CMOS sensors, on the other hand, offer faster readout speeds and lower power consumption, making them more common in modern digital cameras. The presence of color filters, such as the Bayer filter array, determines how the sensor records different colors. Each pixel on the sensor is covered by a red, green, or blue filter, and the camera's image processor combines these to produce a full-color image. Advances in sensor technology, such as backside illumination (BSI) and dual gain architecture, enhance low-light performance and dynamic range, contributing to better color fidelity in challenging lighting conditions. The sensor's bit depth and color processing algorithms also impact the final color quality, with higher bit depths allowing for more nuanced color gradations and smoother transitions.
7. White balance (Liu et al., 1995): adjusting the white balance setting on a camera is essential for compensating for different lighting conditions to ensure accurate color reproduction. White balance settings adjust the camera's color sensitivity to match the color temperature of the light source, measured in Kelvin (K). For example, daylight has a color temperature of around 5,500 K, while tungsten light is warmer at around 3,200 K. Incorrect white balance settings can lead to color casts, where images appear too warm (yellow/orange) or too cool (blue). Most modern cameras offer automatic white balance (AWB) modes that attempt to neutralize these color casts based on the scene's lighting. However, manual white balance adjustments or using presets for specific lighting conditions (e.g., daylight, cloudy, fluorescent) can yield more accurate results. Advanced photographers often use custom white balance settings or white balance bracketing to achieve precise color accuracy. Additionally, post-processing software allows for further white balance adjustments, providing flexibility to correct any color imbalances after the photo is taken. Understanding and controlling white balance is crucial for maintaining color fidelity and achieving the desired mood and tone in photographs.

Now that we have contextualized the color applied to the photography, we are going to delve into the methodology used to determine which photo is fake or not. The process involves a series of analytical steps designed to scrutinize various aspects of the images, using both qualitative and quantitative techniques to identify signs of manipulation or artificial creation.

Our methodology for determining whether a photo is fake or not involves a comprehensive examination of color distribution, luminance histograms, chromaticity coordinates, metadata, and advanced machine learning techniques. By integrating these various analytical tools, we can systematically and reliably identify signs of image manipulation, ensuring a thorough and accurate assessment of each photograph's authenticity.

### 3.2 Look Up Tables

The most general definition of a color Look-Up Table (LUT) (Monga et al., 2012) is a data structure that maps a multidimensional input color variable to another multidimensional output color variable by storing a selectively sampled subset of all possible color inputs (and corresponding outputs) from a multidimensional space. The term “selectively sampled” is crucial here. Well-known device color spaces such as RGB and CMYK, as well as perceptually significant device-independent color spaces such as CIELAB, are of three dimensions, with each dimension requiring a bit depth of eight bits or higher for adequate representation. An 8-bit-per-channel full-resolution LUT in these spaces can demand a substantial amount of storage, often reaching gigabytes for a comprehensive CMYK CIELAB LUT. For most applications, processor RAM and cache memory constraints make the storage of such full-resolution LUTs impractical.

Moreover, the dimensionality of color devices is likely to increase in the future with the adoption of more than three or four primary colors, such as in six-colorant and higher printing technologies. Consequently, practical color LUTs are constructed with a sparser sampling of the input color space, where the sampled values, known as “nodes,” create a geometric lattice or tessellation that divides the multidimensional input color space into a series of subvolumes. The LUT transformation involves three key steps:

1. Identifying the subvolume to which an input color belongs.
2. Retrieving the nodes corresponding to that subvolume.
3. Interpolating among these node values to generate the output color value. Numerous efficient interpolation techniques exist to facilitate this process.

Applied to this research, each generated image is subjected to the application of various advanced color filters, known as Look-Up Tables (LUTs), for the purpose of adjusting the color palette and overall tone of the image. A LUT (Majumder et al., 2000) is a data structure that maps a multidimensional input color variable to another multidimensional output color variable, storing an intelligently sampled subset of all possible input colors and their corresponding outputs in a multidimensional space. This intelligent sampling allows complex color transformations to be performed efficiently and accurately.

The application of these LUTs is carried out to find the filter that best contributes to the believability of the image. Each LUT contains precomputed values necessary to transform the colors of the original image, allowing for quick and consistent adjustments without the need for complex real-time calculations. This is especially useful in processes where multiple color variations need to be evaluated to determine which is the most suitable.

The key feature of LUTs in this context is their ability to make precise and varied color adjustments. By applying different LUTs to an image, various aesthetics and moods can be explored, from warm, saturated tones to cool, desaturated palettes. The use of LUTs makes it easy to quickly and effectively compare these adjustments, allowing you to select the filter that most enhances the authenticity and visual impact of the image.

In practical terms, the process of applying a LUT to an image involves three main steps: first, the subregion of the input color space corresponding to each pixel in the image is identified; second, the LUT nodes corresponding to this subregion are retrieved; and third, these node values are interpolated between to produce the output color value. This interpolation ensures that even with relatively sparse sampling of the color space, a smooth and accurate color transition is obtained in the final image.

### 3.3 Measuring the differences between LUTs

**Figure 2:**
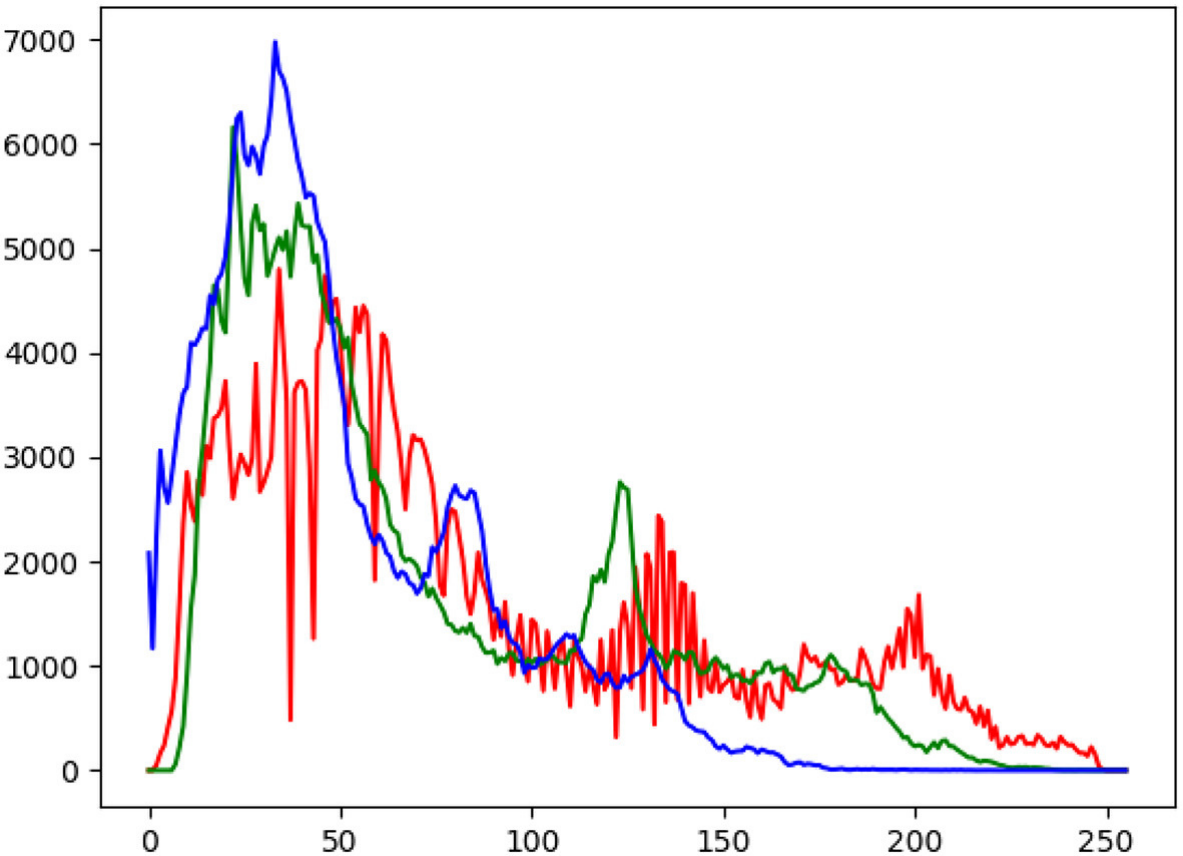
Example histogram like we have used to distinguish between the fake and the real.

This section covers how to describe an image through LUTs. It can be described with histograms. These histograms are the representation of the intensity of each pixel. We are going to show the representation of each color channel. Figure 2 shows an exampling showing its intensity, in which three main color channels show its intensity. The fake images will show an irregular patron due to the artificial luminance, but we cannot assume that it is not real at 100%.

The purpose of these histograms is to qualitatively analyze the luminance of each pixel of the image, allowing one to visually assess the distribution of light and dark areas, which is crucial in understanding the visual characteristics of an image. In the context of image analysis, examining the luminance histogram helps identify patterns, anomalies, and consistency in brightness levels. Assuming the photos were real, the luminance would be expected to show a homogeneous bias throughout the sample, indicating a relatively uniform distribution of light and dark areas across the image, which suggests no unusual or artificial lighting anomalies.

The analysis of luminance through histograms provides a qualitative approach to verify the authenticity and quality of images, as significant deviations from typical real photographs may indicate artificial creation or alteration, and smooth, balanced luminance distributions suggest high-quality images. By assessing the distribution of luminance across each pixel, one can gain insight into the authenticity, quality, and visual coherence of the images, making this method particularly useful for distinguishing between real photographs and artificially generated images, as well as identifying any potential manipulations or defects in the visual content.

### 3.4 Generation of images

**Figure 3:**
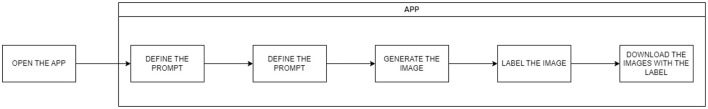
Diagram of how the application for generating war images using generative AI looks like.

The image creation process involves generating images using an artificial intelligence model. Figure 3 shows each of the steps schematically, which will be explained one by one below.

First, a prompt is defined to describe the scene to be generated. This prompt serves as a detailed textual description that guides the AI model in creating the desired image. In this case, the prompt outlines a realistic war scene, specifying elements such as destroyed buildings, columns of smoke rising into the sky, and visible flames among the ruins. The prompt emphasizes a highly detailed and realistic photographic style, ensuring that the generated images are as lifelike as possible. This detailed description is essential for the AI model to accurately visualize and create the scene.

The process uses a loop to generate ten images based on this prompt. By iterating through a loop, the code repeatedly instructs the AI model to create new images, each time using the same detailed prompt. A pipeline within the code takes this prompt and generates an image with specified dimensions (512 × 768 pixels). This indicates the utilization of a sophisticated image generation model like DALL-E, Stable Diffusion, or a comparable technology. These models are crafted to understand textual descriptions and generate matching images, using extensive training on large datasets of images and text to attain high precision and detail in the results.

For each generated image, a random name is created for both the image and its containing folder. This step ensures that each image has a unique identifier, preventing any confusion or duplication. Randomly generated names help maintain an organized structure, especially when dealing with multiple images. The folders are specifically created to store these images, with each image saved in its uniquely named folder. This organizational approach helps in managing the generated content efficiently, making it easier to locate and use each image as needed.

Key components of this process include the generation pipeline, which is likely a pre-trained AI model capable of creating images from textual descriptions. These pretrained models have been extensively trained on diverse datasets, enabling them to understand and visualize complex scenes described in text. The prompt plays a crucial role, as it provides a comprehensive and detailed description of the scene that the model needs to generate. The use of a loop allows for the efficient creation of multiple images, all based on the same prompt but saved with different names to ensure proper organization.

The context suggests that the process is designed to be executed in Google Colab, a popular platform for running Python code and machine learning models in a cloud-based environment. Using Google Colab provides the computational power needed to run advanced AI models, along with the convenience of cloud storage. The integration with Google Drive further enhances this setup, allowing for easy saving and organizing of the generated images. This setup makes it accessible and efficient for users to manage their projects and data.

A detailed prompt and an image generation pipeline are used to create multiple realistic images of war scenes. Each image is saved in a uniquely named folder, ensuring proper organization and management of the generated content.

## 4 Serverless AI architecture

A serverless design is utilized, making use of tools from different cloud platforms like Microsoft Azure, Google Cloud, and, in this instance, Amazon Web Services (AWS). This method makes use of existing computing resources, eliminating the need for local resources that require maintenance, initial investment, and specific usage to avoid damage. AWS Lambda is selected for various reasons, with cost-effectiveness being the most important factor. AWS Lambda enables the deployment of a software model that includes generative artificial intelligence, which has been trained to produce war imagery similar to fake news. Additionally, this computational capability is utilized for parallel and concurrent computation.

**Figure 4:**
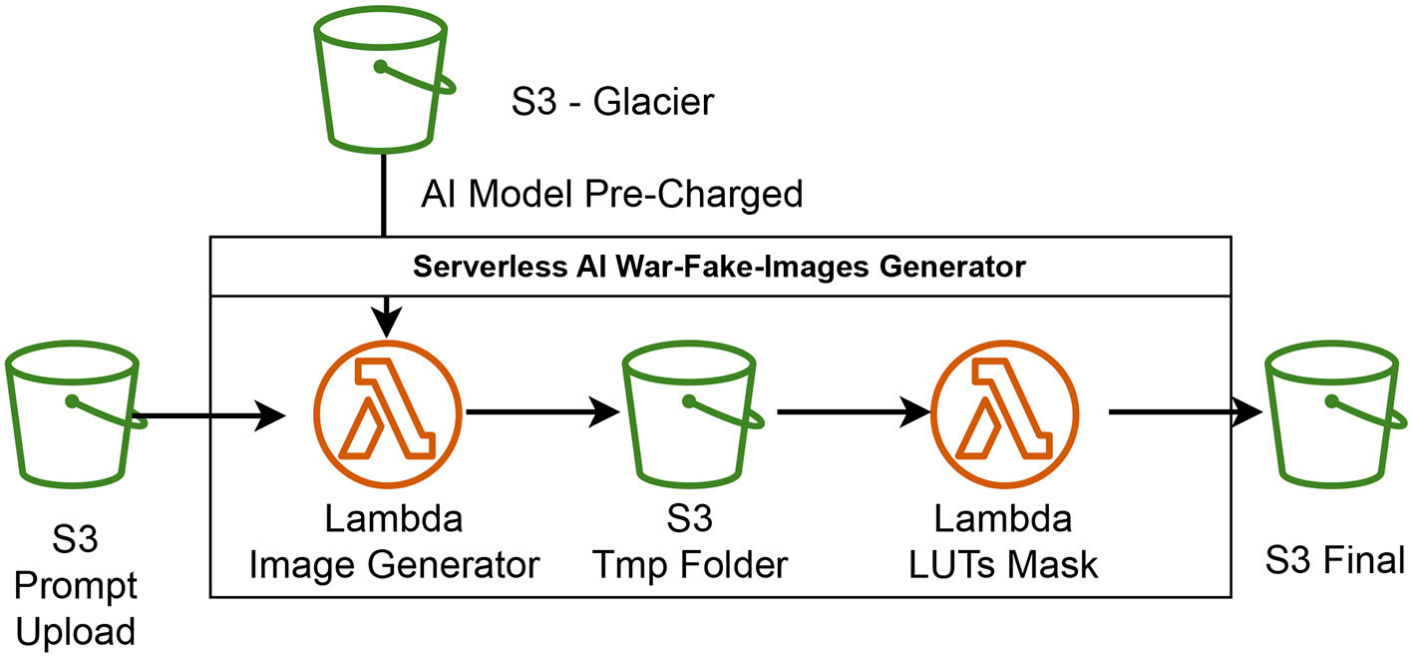
Summary of the serverless AI framework used to create counterfeit war news images. This framework utilizes AWS Lambda for concurrent processing, adopting a serverless methodology to effectively produce a substantial collection of images.

As demonstrated in the serverless architecture (Figure 4), the use of AWS Lambda allows the parallel and simultaneous execution of thousands of functions, significantly enhancing the efficiency of the generation of fake news images. It is demonstrated that when a program is executed on a local computer, attempts can be made to execute it once per core, once per thread, or even on the graphical processing unit for vector computation. However, such executions are limited by the computing capacity of the local computer. With AWS Lambda, it is possible to load and execute this program up to a thousand times in parallel and simultaneously without intercommunication between programs. Consequently, a thousand distinct images can be generated almost instantaneously. The cost-effectiveness of executing a thousand functions in Lambda is equivalent to executing a single function, as charges in Lambda are based on computing capacity used every thousand executions.

Furthermore, an S3 container is introduced, similar to a cloud storage file, where a *txt* file with desired image attributes for generation. This triggers an S3 container event that executes the Lambda function a thousand times with the provided prompt. Automatically, Lambda generates an image using the pre-trained Stable Diffusion model, which is freely downloadable and preloaded in another cold storage S3 for quicker execution. The generated images are then stored in an S3 folder, with each original image from Stable Diffusion being further processed by another Lambda function applying selected color LookUp Tables (LUTs). This process produces ~50,000 images with different color tones and shapes in less than a minute, which are also stored in an S3 container.

To minimize costs, once the images are downloaded from the S3 container, all temporary files and elements in the S3 containers are deleted, except for the preloaded Stable Diffusion file in cold storage. This serverless architecture efficiently and potentially hazardously creates ~50,000 fake war news images in less than two minutes. For analysis of this architecture and the amalgam of colors from the LUTs, a manual examination of each image in the dataset is required. This dataset, slightly reduced to about 700 images, is curated following color patterns, histograms, and similarities with real images, both fake news and photographic, for an exhaustive discussion analysis.

**Figure 5:**
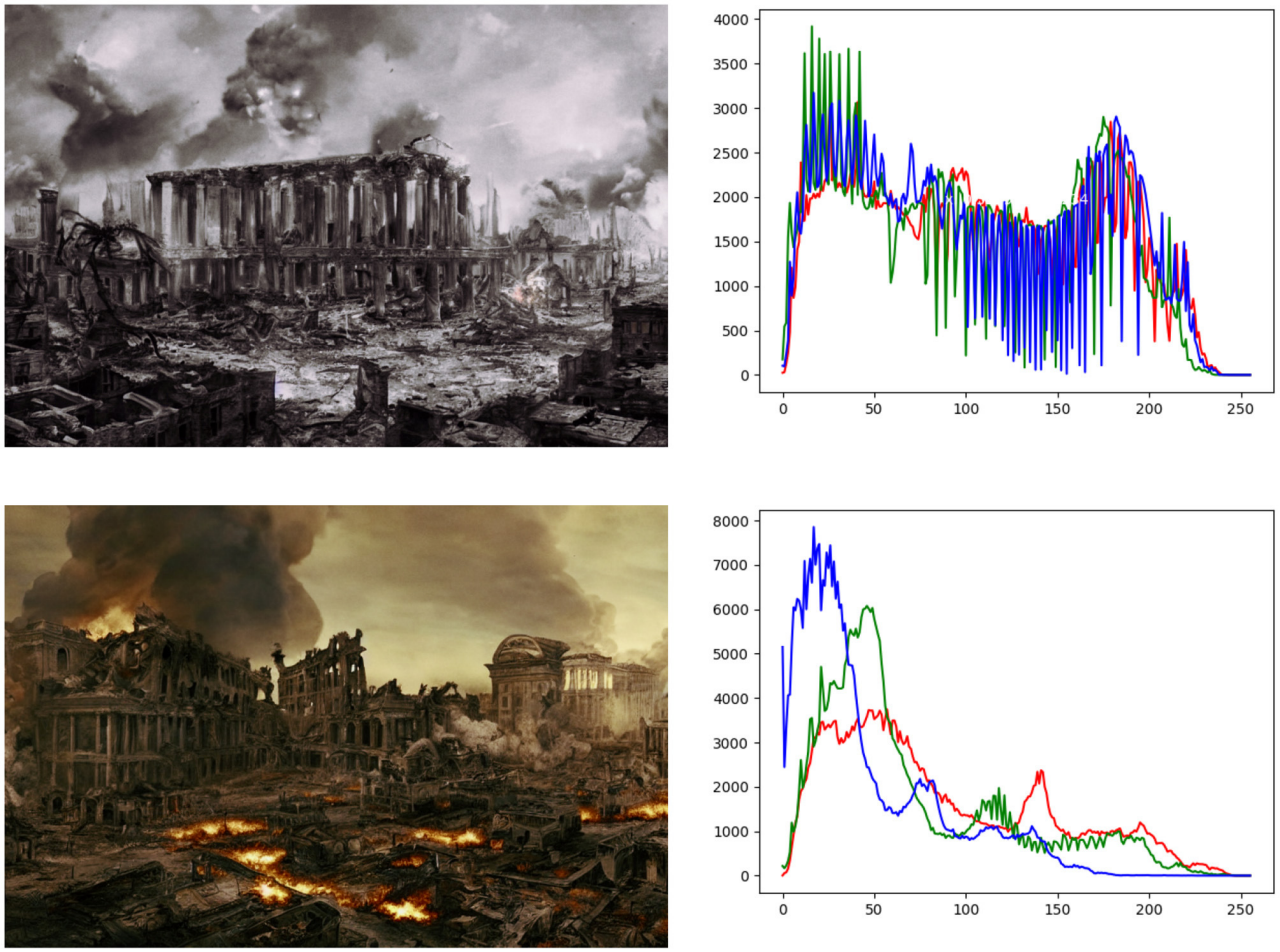
Histograms and representative images generated using the serverless AI architecture. This figure illustrates the variety of color profiles applied through LUTs and the resulting diversity in the perceived authenticity of the generated images.

Additional examination of the produced images and their color histograms (Figure 5) offers valuable insights into the impact of color variations on the perceived authenticity of these images. This analysis contributes to the ongoing discourse on the ethical consequences of AI-generated fake news. The images generated in this study aim to mimic historical war visuals rather than replicate present-day real-world conflicts. This highlights the potential risks associated with serverless computing and artificial intelligence in spreading deceptive information. The purpose of analyzing the dataset is to investigate the factors that contribute to the perceived realism of an image when different LUTs are applied. The goal is to gain insights into how an image can appear more genuine to newspapers, social networks, and the public. This exploration also involves examining the possible societal, political, and economic consequences that could arise if this technology becomes widely accessible.

## 5 Discussion

In this article, an artificial imager has been developed using LUTs and AI. The role of LUTs plays a fundamental role, since, by creating filters it makes the images more realistic. These images will be evaluated by histograms to assess their veracity. The development of this methodology could validate a way to verify if the images that are posted on social networks are false. The use of serverless technology makes it possible to generate these images in seconds and identify if they are true in seconds as well. Since the same system generates both images and histograms in a short time. It opens a new avenue of research for the creation of a verifier.

## 6 Conclusions

In this study, it has been proposed to create a fake warfare image generator based on LUTs. It has had two approaches, one for the colorimetric study of the different images generated to assess if they could be differentiated from the fake ones, and another one, where it is explained how the application and serverless computing are able to generate these images from already trained sets. The use of these LUTs has allowed us to create images that can be passed off as real. The generation of this type of image on social networks can have a social and ethical impact, which can affect the development of war conflicts. As seen in the literature, there is already a use of false images for the creation of fake news today, but with this tool, images of nonexistent wars could be generated, and a new collective memory could be created. The potential of AI has not yet reached its ceiling in this aspect. By combining serverless technology and histogram rating, it would be possible to create a “white box” where the user could see why these generated images are true or false. The use of serverless makes this possible in seconds, because of its structure and the structure of the code itself. It has been demonstrated through the use of histograms that it is possible to differentiate a false image from a real one by its homogeneity. In the future, this false image detection could be improved by using other color-based techniques to differentiate false images from true images.

## Data Availability

The raw data supporting the conclusions of this article will be made available by the authors, without undue reservation.
